# RIPK1–RIPK3–MLKL-Associated Necroptosis Drives *Leishmania infantum* Killing in Neutrophils

**DOI:** 10.3389/fimmu.2018.01818

**Published:** 2018-08-14

**Authors:** Laiana A. Barbosa, Paloma P. Fiuza, Letícia J. Borges, Fellipe A. Rolim, Mayara B. Andrade, Nivea F. Luz, Graziele Quintela-Carvalho, Jonilson B. Lima, Roque P. Almeida, Francis K. Chan, Marcelo T. Bozza, Valeria M. Borges, Deboraci B. Prates

**Affiliations:** ^1^Universidade Federal da Bahia, Salvador, Brazil; ^2^Laboratório de Inflamação e Biomarcadores, Instituto Gonçalo Moniz, Fundação Oswaldo Cruz, Salvador, Brazil; ^3^Instituto Federal de Educação, Ciência e Tecnologia Baiano, Santa Inês, Brazil; ^4^Centro de Ciências Biológicas e da Saúde, Universidade do Oeste da Bahia, Barreiras, Brazil; ^5^Departamento de Medicina, Universidade Federal de Sergipe, Aracaju, Brazil; ^6^Department of Pathology, Immunology and Microbiology Program, University of Massachusetts Medical School, Worcester, MA, United States; ^7^Departamento de Imunologia, Universidade Federal do Rio de Janeiro, Rio de Janeiro, Brazil; ^8^Departamento de Biomorfologia, Instituto de Ciências da Saúde, Universidade Federal da Bahia, Salvador, Brazil

**Keywords:** *Leishmania infantum*, neutrophils, necroptosis, cell death, RIPK3, mixed lineage kinase domain-like, caspase-8

## Abstract

Necroptosis is a pro-inflammatory cell death, which happens in the context of caspase-8 inhibition, allowing activation of the receptor interacting protein kinase 1–receptor interacting protein kinase 3–mixed lineage kinase domain-like (RIPK1–RIPK3–MLKL) axis. Recently, necroptosis has emerged as a key component of resistance against pathogens including infected macrophage by *Leishmania infantum*, the ethiologic agent of Visceral leishmaniasis (VL). VL is the most severe form of Leishmaniasis, characterized by systemic inflammation and neutropenia. However, the role of neutrophil cell death in VL has not been characterized. Here, we showed that VL patients exhibited increased lactate dehydrogenase levels in the serum, a hallmark of cell death and tissue damage. We investigated the effect of necroptosis in neutrophil infection *in vitro*. Human neutrophils pretreated with zVAD-fmk (pan-caspase inhibitor) and zIETD-fmk (caspase-8 inhibitor) increased reactive oxygen species (ROS) level in response to *Leishmania* infection, which is associated with necroptotic cell death. MLKL, an important effector molecule downstream of necroptosis pathway, was also required for *Leishmania* killing. Moreover, in absence of caspases-8, murine neutrophils displayed loss of membrane integrity, higher levels of ROS, and decreased *L. infantum* viability. Pharmacological inhibition of RIPK1 or RIPK3 increased parasite survival when caspase-8 was blocked. Electron microscopy assays revealed morphological features associated with necroptotic death in *L. infantum* infected-neutrophils pretreated with caspase inhibitor, whereas infected cells pretreated with RIPK1 and RIPK3 inhibitors did not show ultra-structural alterations in membrane integrity and presented viable *Leishmania* within parasitophorous vacuoles. Taken together, these findings suggest that inhibition of caspase-8 contributes to elimination of *L. infantum* in neutrophils by triggering necroptosis. Thus, targeting necroptosis may represent a new strategy to control *Leishmania* replication.

## Introduction

Visceral leishmaniasis (VL) is a neglected tropical disease, caused by protozoan parasites of the genus *Leishmania* and is transmitted by the *phlebotomine* sandfly bite. The number of new VL cases worldwide each year is currently estimated at 300,000. *Leishmania infantum* is the etiological agent of VL in Brazil. VL is the most severe form of Leishmaniasis, which causes high morbidity and mortality in affected communities if left untreated (1, 2). Clinically, VL is a chronic infectious disease characterized by fever, weight loss, splenomegaly, hepatomegaly, anemia, cachexia, hematological alterations, and spontaneous bleeding (3, 4). Notably, neutropenia is one of the main laboratorial characteristics of patients with VL (5).

Neutrophils are the first cells recruited to the *Leishmania* infection site and can efficiently phagocytose parasites during the first hours of infection (6). Even though macrophages are the preferential host cell for *Leishmania* parasites in the chronic phase of the disease, neutrophils can also exert varied functions in the context of leishmaniasis. The role of neutrophils in leishmaniasis is controversial, as they can be protective or deleterious depending on the parasite species and the host (7–11). Concerning human VL, the role of neutrophil is also poorly characterized. It was proposed that dysfunctional neutrophils contribute to disease severity and systemic inflammatory response characteristic of VL (12). Recent studies show that neutrophils may contribute to immunosuppression in subjects with active VL (13). HLA-DR^+^ neutrophils from VL patients do not stimulate T-cell proliferation, but they do express higher programmed cell death ligand-1 (13). Moreover, the neutrophil effects on *Leishmania* survival have also been associated with the development of an immune response after the initial stages of infection where cell death pathways can account for a pro- or anti-inflammatory microenvironment in the host (6, 8).

Necroptosis is a regulated form of cell death morphologically characterized by cell and organelle swelling, which ultimately culminates in loss of plasma membrane integrity (14, 15). Molecularly, receptor interacting protein kinases 1 and 3 (RIPK1 and RIPK3, respectively) and mixed lineage kinase domain-like (MLKL) are essential regulators of necroptosis that can be triggered by distinct signals including those involved in apoptosis (16–19). In contrast to necroptosis, apoptosis is an immunologically silent cell death characterized by maintenance of cell integrity that occurs in the presence of caspases. Caspase-8 mediates apoptotic cell death by cleaving and activating downstream caspases, such as caspase-3 and -7. The activation of RIPK1 is an upstream event of necroptosis. When caspase-8 is inhibited, RIPK1 promotes necroptosis by interacting with RIPK3, which mediates the phosphorylation of MLKL, which forms pore in the plasma membrane, promoting cell lysis (20–22). Moreover, necroptosis is an inflammatory cell death that contributes to innate immunity in both humans and mice by killing cells infected by pathogens (23–27). Viral, bacterial, and parasitic infections provoke release of danger signals and, consequently contribute to alert the immune system (18, 20, 21, 28). TNF-induced necroptosis requires RIP kinase activation and caspase-8 inhibition, which controls viral replication (20). More recently, our group showed the role of necroptosis in *Leishmania* infection (28). Using human and mouse macrophages, we identified that RIPK1 and mitochondrial phosphatase phosphoglycerate mutase family member 5 (PGAM5) are two novel host factors that control *Leishmania* replication through distinct mechanisms. PGAM5 promotes optimal IL-1β production, which in turn stimulates nitric oxide (NO) production, whereas RIPK1 regulates *Leishmania* replication independent of IL-1β (28).

In the present study, we show that inhibition of caspase-8 controls *Leishmania infantum* replication inside both, human and murine neutrophils by promoting cell membrane damage and limiting parasite replication. *Leishmania* infection in the presence of caspase-8 inhibition is marked by increased RIPK3 and MLKL expression by human neutrophils. Inhibition of MLKL reduced cell death and restored parasite replication, indicating that necroptosis is active and facilitates human neutrophil control of parasite replication. Under the same condition of caspase-8 inhibition, murine neutrophils display loss of plasma membrane integrity and formation of reactive oxygen species (ROS), suggesting a pro-inflammatory cell death profile. In addition, specific inhibition of RIPK1 or RIPK3 in murine neutrophils reversed parasite killing caused by caspase inhibition. Importantly, pretreatment of neutrophils with zVAD-fmk followed by *L. infantum* infection revealed morphological features of necroptosis in these cells by electron microscopy, whereas addition of the RIPK1 kinase inhibitor Nec-1 or the RIPK3 kinase inhibitor GSK’872 increased *L. infantum* viability in murine neutrophils. Collectively, our results point to a novel and beneficial role of neutrophils in the control of *Leishmania* replication through necroptosis induced by caspase-8 inhibition.

## Results

### Circulating Levels of Lactate Dehydrogenase (LDH) Are Augmented in Patients With VL

**Figure 1:**
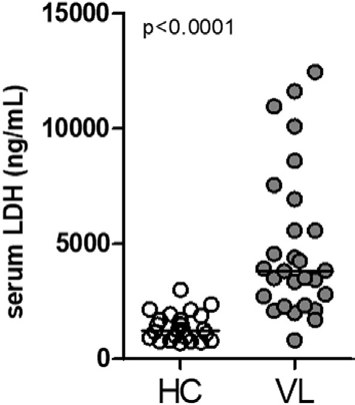
Circulating levels of lactate dehydrogenase (LDH) in patients with visceral leishmaniasis (VL). LDH levels from serum sample obtained from patients with VL (*n* = 33) and healthy controls individuals (HC; *n* = 25) from an endemic area in the Northeast of Brazil was estimated by colorimetric assay (28, 31). Mann–Whitney *U* test was used to verify statistical difference between VL and HC individuals. Circles represent individual values. Black bars represent median values.

Lactate dehydrogenase is a systemic biomarker of tissue/cell death damage related to necroptosis in inflammatory diseases (29, 30). Here, we evaluated the circulating levels of LDH in serum samples from patients with classical VL manifestation before anti-leishmanial therapy and non-infected subjects from the same endemic region of northeast of Brazil (endemic controls) (28, 31). These VL patients showed high plasma LDH levels compared with healthy controls (HC) (*P* < 0.0001) (Figure 1).

### Caspase-8 Inhibition Induces Damage in Human Neutrophils Infected by *L. infantum*

**Figure 2:**
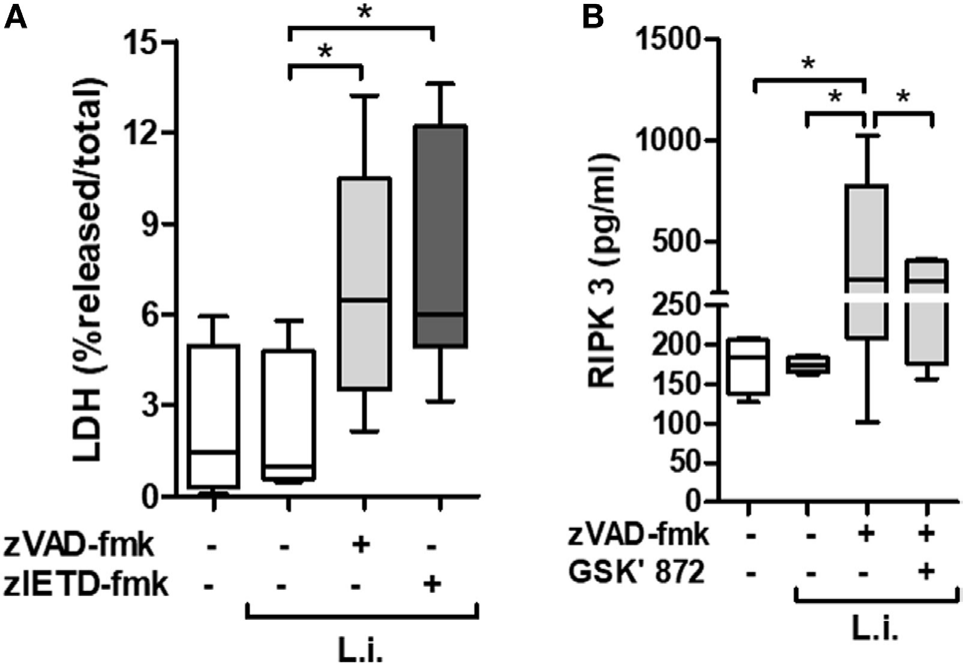
zVAD-fmk and zIETD-fmk treatment induces cell damage in *Leishmania infantum*-infected-human neutrophil. Human neutrophils from health donors (*n* = 6) were pretreated with zVAD-fmk (100 µM) or zIETD-fmk (100 µM) for 30 min. After that, cells were infected with *L. infantum* stationary promastigotes (5 parasites:1 neutrophil). **(A)** 1 h after *in vitro* infection LDH release from damaged cells was measured in supernatant by colorimetric assay. **(B)** Receptor interacting protein kinase 3 (RIPK3) concentrations in cell lysates was measured by ELISA, 3 h after infection in the presence or not of RIPK3 inhibitor (GSK’872, 3 µM). Data shown are from a single experiment representative of three independent experiments. Asterisk indicates significant differences assessed using the Kruskal–Wallis non-parametric test with Dunn’s post-test. *P < 0.05; Abbreviations: L.i., *Leishmania infantum*; LDH, lactate dehydrogenase.

Neutropenia is one of the main clinical characteristics of patients with VL (5). To better explore the association between necroptotic cell death and neutrophil infection by *Leishmania*, we employed an *in vitro* system using primary human neutrophils previously treated with caspases inhibitors, and then infected by *L. infantum* stationary promastigotes (Figure 2). Using the pan-caspase and caspase-8-specific inhibitors, zVAD-fmk and zIETD-fmk, respectively, we detected a significant increase in LDH levels in infected neutrophil culture supernatant, indicating cell damage by loss of plasma membrane integrity (Figure 2A).

**Figure 3:**
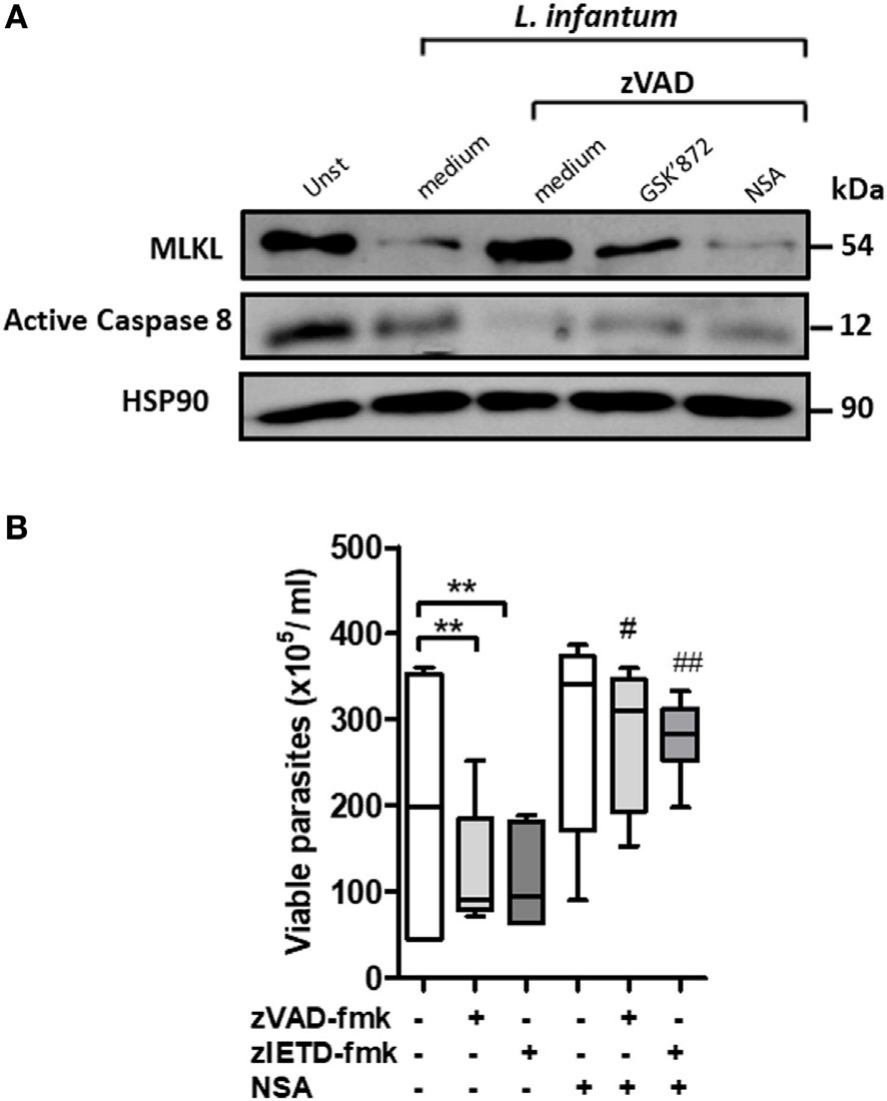
Inhibition of mixed lineage kinase domain-like (MLKL) reverses *Leishmania infantum* killing induced by blockage of caspase-8 in human neutrophils. Human neutrophils from health donors (*n* = 6) were pretreated with zVAD-fmk (100 µM) or zIETD-fmk (100 µM) for 30 min. After that, cells were infected with *L. infantum* stationary promastigotes (5 parasites:1 neutrophil) in the presence or not of necrosulfonamide (NSA) (10 µM) and/or GSK’872 (3 µM). **(A)** Caspase-8 and MLKL expression as detected by western blotting. **(B)** Neutrophils infected with *L. infantum* promastigotes followed by cultivation at 26°C and viable promastigotes counts after 1 day. Data shown are from a single experiment representative of three independent experiments. Asterisk and hash indicate significant differences assessed using the Kruskal–Wallis non-parametric test with Dunn’s post-test. **P < 0.01; ^#^*P* < 0.05, ^##^*P* < 0.01. Statistical comparisons between control groups (white bars) and groups that received treatment with zVAD-fmk/zIETD-fmk are shows as *. Statistical comparisons between zVAD-fmk/zIETD-fmk groups and groups that received NSA after treatment with zVAD-fmk/zIETD-fmk are shows as ^#^.

In order to investigate the presence of specific molecules of necroptotic cell death pathway, we examined the receptor interacting protein kinase 3 (RIPK3) production by *L. infantum*-infected neutrophils in the context of caspases inhibition (Figure 2B). Cell extracts from infected neutrophils in the presence of zVAD-fmk, showed increased RIPK3 release. Interesting, a pharmacological inhibitor of RIPK3, GSK’872, was able to reduce its production (Figure 2B). In addition, we analyzed the effective inhibition of caspase-8 in *L. infantum*-infected neutrophils pretreated with zVAD-fmk (Figure 3A). Taken together, these results suggest that *L. infantum-*infected human neutrophil undergo necroptosis when caspases, especially caspase-8, are inhibited.

### Human Neutrophils Control *L. infantum* Viability in an MLKL-Dependent Manner

Next, we investigated the role of MLKL on *Leishmania* survival inside human neutrophils. MLKL is an important downstream effector molecule in the necroptosis pathway. MLKL interacts with activated RIPK3, resulting in cell lysis, a hallmark of necroptosis (32–34). Necrosulfonamide (NSA) is an effective pharmacological inhibitor of human MLKL (33, 35). First, MLKL expression on *L. infantum*-infected neutrophil was analyzed by immunoblot (Figure 3A). Immunoblots revealed that the MLKL was increased following *L. infantum* infection when caspases are inhibited and its reduction in the presence of RIPK3 and MLKL necroptotic inhibitors, GSK’872 and NSA, respectively (Figure 3A). Moreover, human neutrophils pretreated with the caspases inhibitors zVAD-fmk or zIETD-fmk controlled *L. infantum* replication (Figure 3B). In the presence of NSA, human neutrophils showed a significant increase in the parasite burden when compared with neutrophils pretreated only with caspase inhibitors (Figure 3B). Taken together, these results suggest that the RIPK3–MLKL-dependent necroptosis pathway is active in human neutrophils during *L. infantum* infection in the absence of caspase-8, which contributes to parasite killing.

### Necroptosis Reduces *L. infantum* Viability in Murine Neutrophils

**Figure 4:**
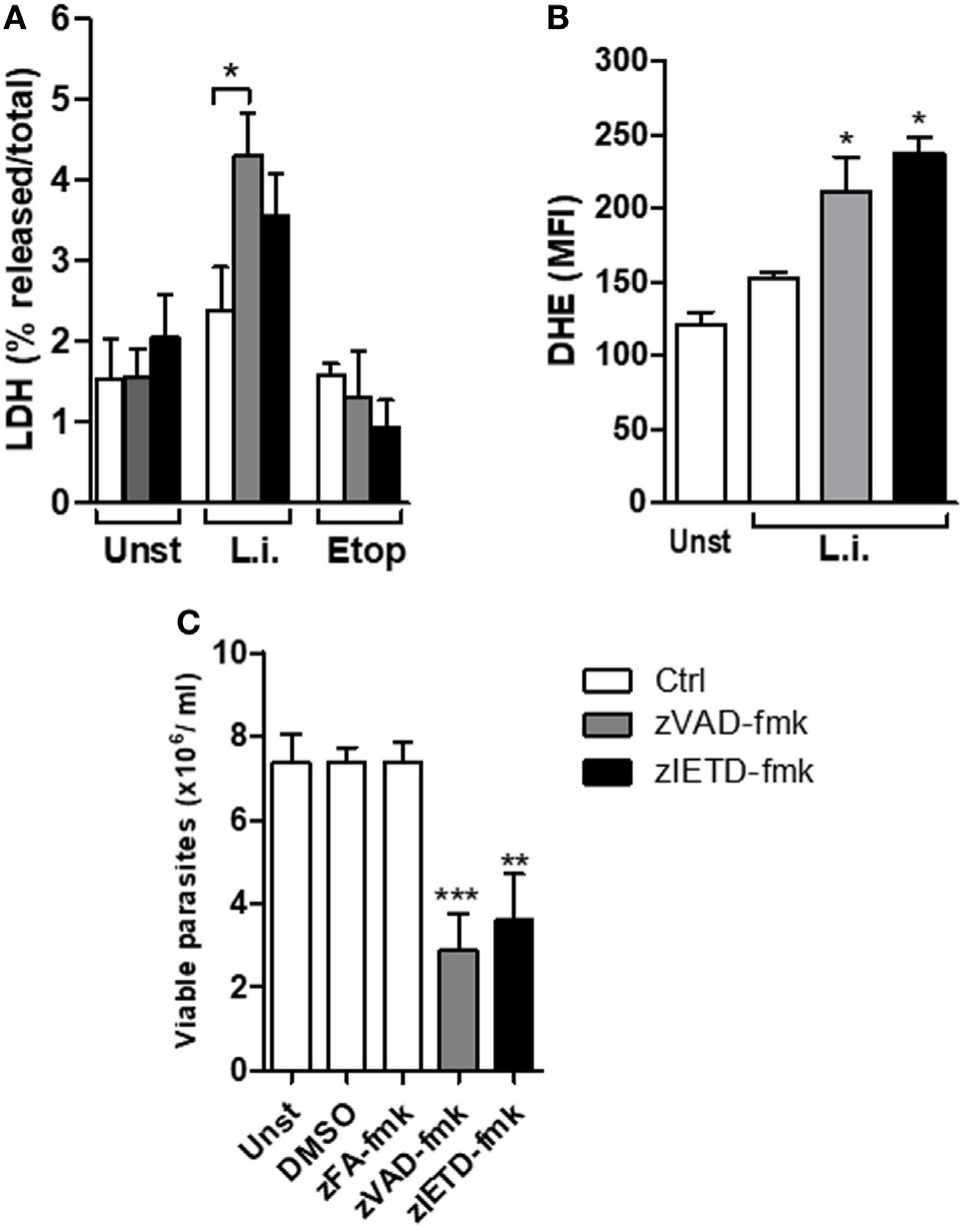
zVAD-fmk and zIETD-fmk treatment induces cell damage, high ROS production, and reduces *Leishmania infantum* parasite viability in murine neutrophils. Inflammatory neutrophils from C57BL/6 mice were obtained after i.p. thioglicolate (3%) injection. Neutrophils (5 × 10^5^/well) were pretreated with zVAD-fmk (100 µM) or zIETD-fmk (100 µM) or zFA-fmk control (100 µM) for 30 min. After that, cells were infected with *L. infantum* stationary promastigotes (5 parasites:1 neutrophil) for 1 h **(A,B)** and 18 h **(C)**. **(A)** Colorimetric assay was performed 1 h after infection to quantitatively measured lactate dehydrogenase (LDH) released into the media from damaged cells as a biomarker for cellular cytotoxicity and cytolysis. **(B)** One hour after infection, neutrophils were incubated with DHE and intracellular ROS production was evaluated by flow cytometry. **(C)** Eighteen hours after infection, neutrophils were followed by cultivation at 26°C and viable promastigotes counts were performed after 1 day. Data shown are from a single experiment representative of three independent experiments. Asterisk indicates significant differences assessed using the Kruskal–Wallis non-parametric test with Dunn’s post-test. *P < 0.05; ** *P* < 0.01; ***P < 0.001. Statistical comparisons between control groups (white bars) and groups that received treatment with zVAD-fmk/zIETD-fmk are shows as *. Abbreviations: Unst, non-infected neutrophils; L.i., *Leishmania infantum*; Etop, etoposide; ROS, reactive oxygen species; DHE, dihydroethidium.

We have previously reported that murine neutrophils undergone apoptosis upon *L. infantum* infection and this effect which was enhanced by saliva of *Leishmania* vector, was correlated with increased parasite load associated with apoptosis death (36). We therefore asked whether blockage of caspases on *L. infantum*-infected neutrophils switch the immunologically silent death pathway from apoptosis to a pro-inflammatory death in these cells. First, we investigated the effect of caspase inhibition in mouse neutrophil viability using the pan-caspase and specific caspase-8 inhibitors, zVAD-fmk and zIETD-fmk, respectively (Figure 4A). In the presence of caspase inhibition, *Leishmania*-induced cell death as measured by increased LDH release was increased in infected neutrophils (Figure 4A). As control, in the presence of etoposide, an apoptosis inducer, there was no increase of LDH release in the presence of caspase inhibition (Figure 4A). In addition, we measured the generation of ROS by infected neutrophils in this system (Figure 4B). Significant increase of intracellular ROS was observed within 60 min of infection when caspases were inhibited by zVAD-fmk, and this effect was further increased with the specific caspase-8 inhibitor zIETD-fmk (Figure 4B).

In order to investigate the impact of the switch apoptotic neutrophil death to necroptosis on *L. infantum* survival, we assessed the *in vitro* parasite viability in the cell cultures (Figure 4C). Similar to human neutrophils, we found a significant decrease in *Leishmania* viability in mouse neutrophils when caspase-8 was inhibited by pretreatment with zVAD-fmk or zIETD-fmk (Figure 4C). To rule out toxic effect of caspase inhibitors on the parasite, we tested whether treatment with zVAD-fmk or zIETD-fmk could directly affect parasite viability. We found that these inhibitors were not toxic to *Leishmania* parasites (Figure S1 in Supplementary material). These data reinforce the results obtained from human neutrophils and suggest that inhibition of caspase, specifically caspase-8, contributes to *L. infantum* killing.

### RIPK1 and RIPK3 Inhibition Abrogates *L. infantum* Killing Induced by Necroptosis

**Figure 5:**
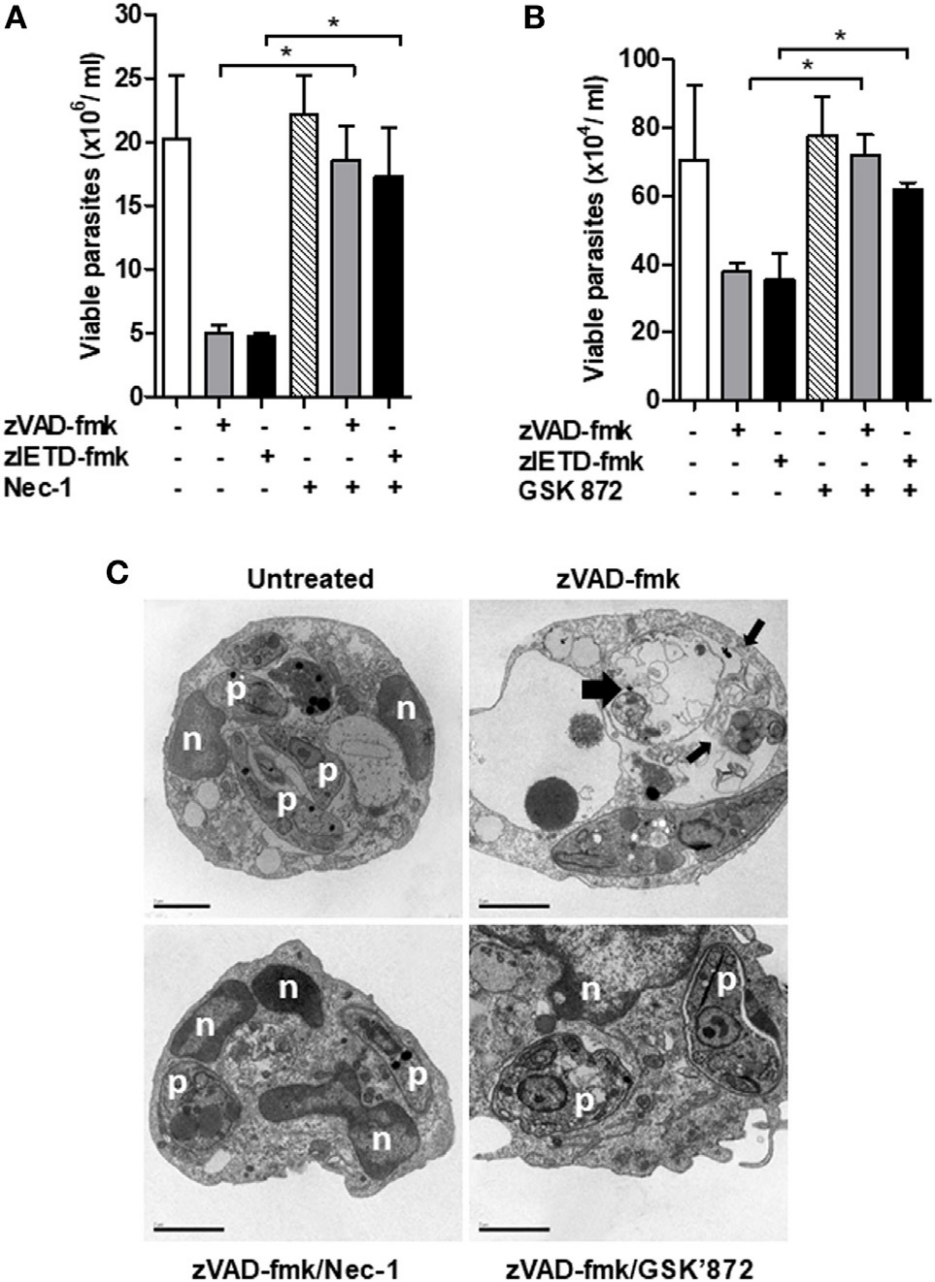
Inhibition of RIPK1 and RIPK3 reverses *Leishmania infantum* killing induced by inhibition of caspase-8. Inflammatory neutrophils from C57BL/6 mice were obtained after i.p. thioglicolate (3%) injection. Neutrophils (5 × 10^5^/well) were pretreated with zVAD-fmk (100 μM) or zIETD-fmk (100 μM) for 30 min. After that, cells were infected with *L. infantum* stationary promastigotes (5 parasites:1 neutrophil) in the presence or not of **(A)** Nec-1 (50 µM, RIPK1 inhibitor) or **(B)** GSK’872 (3 µM, RIPK3 inhibitor) followed by cultivation at 26°C and viable promastigotes counts after 1 day. Data shown are from a single experiment representative of three independent experiments. Asterisk indicates significant differences assessed using the Kruskal–Wallis non-parametric test with Dunn’s post-test. *P < 0.05. **(C)** Representative transmission electron micrographs of inflammatory neutrophils pretreated with zVAD-fmk followed of infection with *L. infantum* stationary promastigotes in the presence or not of Nec-1 and GSK’872. Morphological features of necroptosis in *L. infantum* infected-neutrophils pretreated with zVAD-fmk is reversed by inhibition of RIPK1 and RIPK3. Untreated infected neutrophils (negative control), viable parasites (p), intact nuclei (n). Scale bars = 2 µm.

Interaction between RIPK1 and RIPK3 accounts for the formation of the ripoptosome complex, which is essential for necroptosis activation (26, 37, 38). Usually, this complex is assembled in conditions of caspase-8 inhibition (15, 26). In order to explore the involvement of the axis RIPK1–RIPK3 on *L. infantum* replication in mice neutrophils, we performed infection assays in the presence of necrostatin-1 (Nec-1) or GSK’872, specific inhibitors of RIPK1 and RIPK3, respectively (Figure 5). C57BL/6 neutrophils pretreated with zVAD-fmk or zIETD-fmk controlled *L. infantum* replication (Figures 5A,B). Importantly, the opposite effect was observed when neutrophils were incubated with RIPK1 (Figure 5A) or RIPK3 (Figure 5B) inhibitors. Taken together, these data suggest that the RIPK1–RIPK3 complex is active during *L. infantum* infection in neutrophils when caspases are inhibited, which limited parasite replication.

After these results, we then decided to investigate whether the morphological aspects presented in *L. infantum*-infected neutrophils pretreated with the caspase inhibitor zVAD-fmk corresponds to necroptosis cell death morphology (Figure 5C). Distinct from apoptosis, necroptosis cell death is morphologically characterized by swelling of organelles and plasma membrane rupture (39–42). Transmission electronic microscopy assays revealed that zVAD-fmk pretreated *L. infantum*-infected neutrophils exhibited plasma membrane and organelle rupture (thin black arrows) and dead parasites (thick black arrows) (Figure 5C). Moreover, groups treated with inhibitors of RIPK1 (Nec-1) or RIPK3 (GSK’872) maintained plasma membrane integrity, preserved intracellular content and interestingly, displayed viable *Leishmania* (p) within parasitophorous vacuole, in contrast with neutrophils pretreated with zVAD-fmk only. Combined, these data clearly indicate the participation of neutrophil necroptosis in *L. infantum* killing.

## Materials and Methods

### Ethics Statement

This study was performed with both, human and animal neutrophils. For human, it was carried out in accordance with the recommendations of Institutional Review Board of the Federal University of Sergipe, Brazil with written informed consent from all subjects. All subjects gave written informed consent in accordance with the Declaration of Helsinki. The protocol was approved by the Institutional Review Board of the Federal University of Sergipe, Brazil (license number: 04587312.2.0000.0058). *In vitro* experiments were performed using buffy coats from healthy blood donors at the state blood bank, Salvador, Brazil. For animals, inbred male C57BL/6 mice, aged 6–8 weeks, were obtained from the animal facility of CPqGM-FIOCRUZ (Bahia, Brazil). All experimental procedures were approved and conducted according to the Brazilian Committee on the Ethics of Animal Experiments of the Centro de Pesquisas Gonçalo Moniz—Fundação Oswaldo Cruz (CPqGM-FIOCRUZ, license number: 004/2014).

### Parasites Culture

*Leishmania infantum* (MCAN/BR/89/BA262) parasites were grown at 23°C in hemoflagellate-modified minimal essential medium (HOMEM medium) containing 10% (v/v) HI-FCS and 24.5 mM hemin (BOD incubator). In all experiments, the cultures were used at stationary phase.

### Mouse and Human Neutrophil Cultures

Mouse neutrophils were obtained as described previously (36, 43). Briefly, C57BL/6 mice were intra peritoneally injected with aged 3% thioglycolate (Difco, Detroit, MI, USA) solution. Seven hours after injection, peritoneal lavage was performed using 10 ml RPMI-1640 medium (Invitrogen, Carlsbad, CA, USA) supplemented with 1% Nutridoma-SP (Roche, Indianapolis, IN, USA), 2 mM l-glutamine, 100 U/ml penicillin, and 100 g/ml streptomycin (Invitrogen, Carlsbad, CA, USA). Exudate cells were incubated at 37°C in 5% CO_2_ for 1 h in 250 ml flasks (Costar, Cambridge, MA, USA) to remove adherent cells. Cells on supernatants were then recovered and cell viability was determined by trypan blue exclusion (>95%; data not shown). Nonadherent cells were stained with anti-Gr-1 and Ly-6G to assess neutrophil purity and were subsequently analyzed by flow cytometry using CellQuest software (BD Immunocytometry Systems, San Jose, CA, USA). Gr-1^+^Ly-6G^+^ cells were routinely >95% pure.

Human neutrophils were obtained from blood of healthy donors from Hemocentro do Estado da Bahia (Salvador, Brazil) after donors had given written, informed consent. This approach was approved by the Research Ethics Committee of FIOCRUZ-Bahia. Human neutrophils were isolated by gradient separation with polymorphonuclear medium (PMN) according to the manufacturer’s instructions (Robbins Scientific, Sunnyvale, CA, USA). Briefly, blood collected was added to vials contained PMN medium and then centrifuged for 30 min at 300 *g* at room temperature. Neutrophils were collected and washed three times with saline by centrifugation for 10 min at 200 *g*.

For *in vitro* assays, mice or human neutrophils (5 × 10^5^/well) were cultured in 200 μl RPMI-1640 medium, supplemented with 1% Nutridoma-SP, 2 mM l-glutamine, 100 U/ml penicillin, and 100 g/ml streptomycin in 96-well plates (Nunc, Denmark).

### Necroptosis and *Leishmania* Infection Assays

Neutrophils were infected *in vitro* with *L. infantum* promastigotes stationary-phase at a ratio of 1:2 (neutrophil:parasites). For assays of cell death, mouse and human neutrophils were pretreated for 30 min with zVAD-fmk (100 µM) (R&D Systems, Minneapolis, MN, USA) or zIETD-fmk (100 µM) (R&D Systems, Minneapolis, MN, USA) to block caspase activation before infection. In some experiments, Nec-1 (50 µM), GSK’872 (3 µM), or NSA (10 µM), necroptosis inhibitors (all from Merck Millipore’s Calbiochem^®^, Darmstadt, Germany) were used. DMSO (vehicle) 0.4% (Cayman Chemical; Ann Arbor, MI, USA) was used as control. After 18 h, mouse infected neutrophils, or after 3 h, human infected neutrophils, were centrifuged, supernatants containing noninternalized promastigotes were collected, and medium was replaced by 250 µl Schneider insect medium (Sigma-Aldrich, St. Louis, MO, USA), supplemented with 20% inactive FBS, 2 mM l-glutamine, 100 U/ml penicillin, and 100 g/ml streptomycin. After that, infected neutrophils were cultured at 25°C for an additional 3 days and intracellular load of *L. infantum* was estimated by production of proliferating extracellular motile promastigotes in Schneider medium (43).

### LDH Quantification

Lactate dehydrogenase activity on supernatants from *L. infantum*-cultured neutrophils was measured spectrophotometrically using a commercial LDH Cytotoxicity Detection Kit (Boehringer Mannheim) to access plasma membrane integrity. According to the manufacturer’s instructions, the absorbance was recorded at 490 nm using a microELISA plate reader (490 nm). Blank LDH levels were subtracted from experimental LDH values and total LDH activity was determined by lysing the cells with 1% Triton X. The percentage of LDH release was calculated by [(LDH) sample × 100]/total (LDH).

Serum LDH was measured using an ELISA kit from Wuxi Douglin Sci. (Wuxi, China). Serum of patients with classical VL before leishmaniasis chemotherapy (*n* = 33) and HC (*n* = 25) was obtained from an endemic area in northeastern Brazil. The clinical and epidemiological characteristics of the study population have been previously described in detail (31, 44).

### RIPK3 ELISA Assay

For the quantitative determination of human receptor interacting protein kinase 3 (RIPK3) concentrations in cell lysates we used a Human Receptor-Interacting Serine/Threonine-Protein Kinase 3 (RIPK3) ELISA Kit (CUSABIO). Human neutrophils (10^6^/well) pretreated with for 30 min with zVAD-fmk (100 µM) were infected with *L. infantum* in the presence of GSK’872 (3 µM) as described above. After 3 h, human infected neutrophils were collected, diluted with 1× PBS (pH 7.2–7.4), until cell concentration reached 100 million/ml, and stored overnight at −20°C. After two freeze-thaw cycles to break up the cell membranes, lysates were centrifuged for 5 min at 5,000 × *g*, 4°C and used to RIPK3 ELISA assay according to the manufacturer’s instructions.

### Western Blot

Total cell protein was isolated from pelleted neutrophils using cell lysis buffer. Absolute protein content of lysates was determined by Bradford assay (Bio-Rad, Hercules, CA, USA). Samples were boiled at 95°C for 5 min and then were run on 12% SDS-PAGE gels. Proteins were transferred onto nitrocellulose membranes, blocked with 5% fat-free milk in TBST for 1 h, and detected using rabbit anti-MLKL antibody-N-terminal (Abcam), mouse anti-caspase-8 (Enzo Life Sciences), and mouse anti-Hsp90 (BD Biosciences) monoclonal primary antibodies. Anti-rabbit MLKL, anti-mouse caspase-8, and anti-mouse-Hsp90 secondary antibodies (all from Abcam) were then applied to membrane, which were subsequently incubated with Western Blotting Detection Reagent (Thermo Scientific) and imaged using ImageQuant LAS 4000 System (GE Healthcare).

### Measurement of Intracellular ROS Production

Intracellular ROS detection in *L. infantum*-infected neutrophils cultured was performed using dihydroethidium (DHE) fluorescent probe (Invitrogen, Carlsbad, CA, USA) following analyses by FACS, according to the manufacturer’s instructions. For investigation of ROS production, the purified neutrophil population was analyzed by forward- and side-scatter parameters following application of the DHE probe.

### Transmission Electron Microscopy

Neutrophils were fixed at room temperature for 2 h in 2.5% glutraraldehyde and paraformaldehyde 2% in 0.1 M cacodylate buffer, pH 7.4. Postfixed with 1% OsO_4_, 0.8% potassium ferricianide, 5 mM CaCl2 in 0.1 M cacodylate buffer. Samples were washed, dehydrated in acetone, and then embedded in PolyBed 812 (Polysciences, Inc.) resin. Ultrathin sections were stained with uranyl acetate and lead citrate and examined on a Zeiss109 transmission electron microscope operating at 80 kV.

### Statistical Analyses

Each experiment was performed using at least five mice/group and it was repeated at least three times. *In vitro* assays using human neutrophils were performed with *n* = 6. All results are reported as mean ± SE of representative experiments and were analyzed using GraphPad Prism 5.0 (GraphPad Software, San Diego, CA, USA). Data distribution from different groups was compared using the Kruskal–Wallis test with Dunn’s multiple comparisons, and comparisons between two groups were explored using the Mann–Whitney test.

## Discussion

The physiological role of neutrophils is directed toward the eradication of invading pathogens (45). In leishmanial infections, the role of neutrophils is controversial. Neutrophil can play a positive (46) or negative role (47) in the outcome of the infection. We previously demonstrated the sophisticated interplay between innate immune response and different cell death pathways in *Leishmania* infection (28, 36, 48, 49). Moreover, neutrophils can undergo necroptosis followed by the ligation of adhesion receptors under inflammatory conditions (50, 51).

Most studies have focused on the role of necroptosis in viral and bacterial infections (52) [reviewed in Ref. (53, 54)]. There are very few studies focusing on infectious diseases caused by protozoan parasites. Our group carried out a study investigating the involvement of necroptosis in the control of different *Leishmania* species (28). RIPK1 and PGAM5 are involved in the control of *Leishmania* replication in macrophages. Interestingly, in that study, the control of parasite replication was dependent on RIPK1 kinase activity. Collectively, these data suggest a potential role for necroptosis in the control of *Leishmania* viability by different cell types. Nevertheless, histology sections of wild type, Ripk1^kd/kd^, and *Pgam5*^−/−^ mice infected with *Leishmania amazonensis* revealed tissue inflammation marked by neutrophil infiltration (28), indicating the importance of these cells in the context of *Leishmania* infection. However, the precise mechanisms or molecules involved in this cell death pathway could be distinct in different cells.

Despite the importance of neutrophils in human VL, the role of neutrophil necroptosis upon *Leishmania* infection had not been investigated. Assessment of a biomarker of cell/tissue damage related to inflammatory cell death, revealed high circulating levels of LDH in VL patients. LDH is a systemic biomarker of cell damage that could be related to necroptosis (29, 30). Because inflammatory imbalance and neutropenia are hallmarks of human VL, we investigated the mechanisms involved in neutrophil cell death when caspases are pharmacologically inhibited before infection with *Leishmania infantum*. We found that specific caspase-8 inhibition contributes to *L. infantum* killing by RIPK1–RIPK3–MLKL-dependent necroptosis, in both human and mouse neutrophils.

In the presence of the pan-caspase inhibitor zVAD-fmk, we noticed human and mouse neutrophil cell death with an early release of LDH. We have previously demonstrated that *L. chagasi* (syn. *infantum*) induces mouse neutrophil apoptosis (36), a non-inflammatory programmed form of cell death involving caspases. In the context of caspase inhibition, these serine proteases could switch apoptosis to necroptosis, a pro-inflammatory and regulated form of cell death, characterized by loss of plasma membrane permeability and release of intracellular contents, as LDH. In this regarding, our data obtained with mouse neutrophils combined with our previous data (36) reinforce the possibility of the use of specific pharmacological inhibitors of caspases such as zVAD-fmk to promotes a switch from apoptosis to others types of regulated cell death on mammalian system (55, 56).

Herein, inhibition of human and mouse neutrophil apoptosis by zVAD-fmk or zIETD-fmk reduced the number of viable parasite within those cells. zVAD-fmk is the most commonly used pan-caspase inhibitor and it has been demonstrated to have low cytotoxicity *in vitro* and *in vivo* (57–60). However, zVAD-fmk can induce necrotic cell death in certain cell lines (61, 62). We rule out the possibility of zVAD-fmk being involved in neutrophil necrotic death by performing cytotoxicity assays (data not shown). Moreover, we also analyzed whether there is any cytotoxic effect of zVAD-fmk on *L. infantum* promastigotes. Indeed, we did not observe alteration on *Leishmania* parasites grow curve. Using a specific caspase-8 inhibitor zIETD-fmk, we reinforce the idea that in the absence of caspase-8, *L. infantum*-infected neutrophils cell death switches from apoptosis to necroptosis with a pro-inflammatory profile, represented by increased ROS production. ROS contribute to the execution of necroptosis (41). It has been observed that ROS triggers necroptosis by promoting peroxylation of lipids, proteins, and DNA, or as second messengers in the signaling pathways of death receptors (41, 63).

It is known that superoxide anion $\left({\text{O}}_{2}^{-}\right)$ and NO are two important molecules critical in controlling *Leishmania* infection (64, 65). *L. infantum* replication in macrophages was controlled through distinct mechanisms involving NO and IL-1β (28). Here, we did not find detectable IL-1β in our assays in neutrophils, discarding the possibility of canonical pyroptosis. Neutrophils, monocytes, and macrophages can control parasites by ROS that are produced by the respiratory burst after phagocytosis (66, 67). Moreover, recently our group shows that heme drives oxidative stress-associated cell death in human neutrophils infected by *L. infantum* (49). Also, in hemorrhagic shock models, it was demonstrated that exosomes released from macrophages promote neutrophil necroptosis mainly by NADPH oxidase-derived ROS production within neutrophils (68). Whether ROS are involved in *L. infantum* viability control during neutrophil necroptosis remains to be investigated.

Here, RIPK3 was released extracellularly after treatment with zVAD-fmk. Although RIPK3 is an intracellular protein which acts in programmed cell death pathways, extracellular release of RIPK3 following necroptosis was previously related on plasma and/or culture supernatants (69). Nevertheless, recently it was described that RIPK1 and RIPK3 could be involved in inflammatory process independently of necroptosis induction (70). RIPK1 and RIPK3 pharmacological inhibition was found to restore *L. infantum* growth in murine neutrophils pretreated with zVAD-fmk or zIETD-fmk. We performed *in vitro* assays using pharmacological inhibitors: Nec-1 (RIPK1 inhibitor) or GSK’872 (RIPK3 inhibitor). We came to the conclusion that reduced parasite grown inside neutrophil, in the context of caspase inhibition was due to induction of necroptosis.

Electron microscopy (EM) remains an important qualitative method to detect cell death morphological features. On EM images, necrotic/necroptotic cells display loss of membrane integrity, low cytoplasm density, disintegrated cell membrane, loss of chromatin, increase in cell volume, swelling of organelles, and cellular collapse (53, 71). Herein, EM images revealed morphological features of necroptosis in *L. infantum*-infected neutrophils subsequent to zVAD-fmk treatment. Interestingly, these morphological aspects of necroptosis were prevented by using Nec-1 and GSK’872. Nec-1 is an allosteric RIPK1 kinase inhibitor able to prevent the formation and activation of RIPK1–RIPK3 complexes (17), whereas GSK’872 inhibits specifically RIPK3 phosphorylation (72). Here, we provide evidence that RIPK1 and RIPK3 are activated in *L. infantum*-infected neutrophils in the absence of caspase-8, thereby promoting neutrophil necroptotic death and killing of *L. infantum* parasites.

Recent evidence described that necroptotic cell death occurs upon the assembly of a large, signal-induced multiprotein complex containing RIPK1, RIPK3, and MLKL, namely necrosome (73). Active MLKL either directly or indirectly destabilizes plasma membrane integrity leading to cell swelling, membrane rupture, and DAMPs release (33, 74, 75). Since MLKL inhibitors specific for mouse cells are not available, we tested here the participation of MLKL using human neutrophils treated with the NSA, a human MLKL inhibitor. Indeed, we found that caspase inhibition contributes to the control of parasite viability in neutrophils *via* MLKL. Moreover, as in the process of necroptosis, MLKL functions as a substrate to RIPK3, it seems that RIPK3 inhibition reduced the expression of MLKL and, consequently the necroptotic *L. infantum*-infected neutrophils.

In summary, our data suggest that interference of neutrophil apoptosis by inhibition of caspases contributes to elimination of *L. infantum* parasites, probably by stimulating an inflammatory response associated with RIPK1–RIPK3–MLKL-dependent necroptosis. In this context, targeting neutrophil cell death pathways by necroptosis may be new strategies to treat human VL.

## Ethics Statement

This study was carried out in accordance with the recommendations of Institutional Review Board of the Federal University of Sergipe, Brazil with written informed consent from all subjects. All subjects gave written informed consent in accordance with the Declaration of Helsinki. The protocol was approved by the Institutional Review Board of the Federal University of Sergipe, Brazil (license number: 04587312.2.0000.0058). All experimental procedures using animals were approved and conducted according to the Brazilian Committee on the Ethics of Animal Experiments of the Centro de Pesquisas Gonçalo Moniz—Fundação Oswaldo Cruz (CPqGM-FIOCRUZ, license number: 004/2014).

## Author Contributions

LAB, PF, MA, NL, MB, VB, and DP conceived and designed the study. LAB, PF, LJB, FR, MA, NL, GQ-C, JL, and DP performed the experiments. LAB, PF, LJB, FR, MA, NL, MB, FC, VB, and DP contributed with data analysis. MB, RA, VB, and DP provided materials and infrastructural support. LAB, NL, MB, FC, VB and DP wrote and revised the manuscript.

## Conflict of Interest Statement

The authors declare that they do not have a commercial association that might pose a conflict of interest. The handling Editor declared a shared affiliation, though no other collaboration, with one of the authors MB.
