# Supplementary figures and images for: RIPK1–RIPK3–MLKL-Associated Necroptosis Drives Leishmania infantum Killing in Neutrophils
Source: Front Immunol. 2018 Aug 14;9:1818. doi: 10.3389/fimmu.2018.01818 (PMC6102393; doi:10.3389/fimmu.2018.01818)

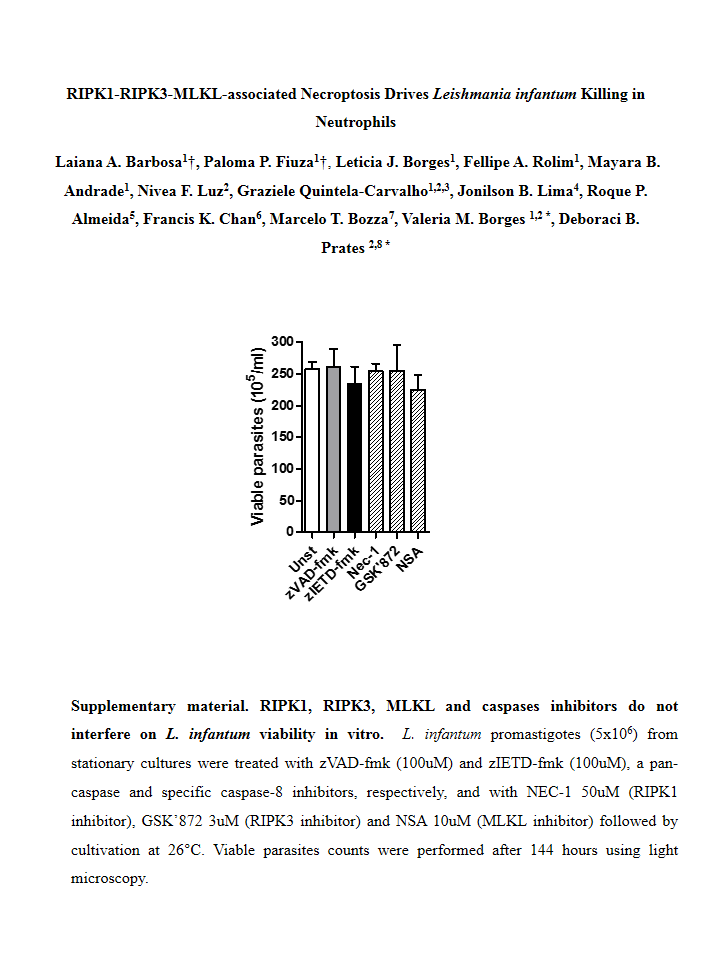

Supplement: Supplementary file 1 [file Image_1.TIF]
